# Interactive Learning for Network Anomaly Monitoring and Detection with Human Guidance in the Loop

**DOI:** 10.3390/s23187803

**Published:** 2023-09-11

**Authors:** Dong Yang, Ze Liu, Songjie Wei

**Affiliations:** School of Computer Science and Engineering, Nanjing University of Science and Technology, Nanjing 210094, China; yangdong21014@163.com (D.Y.);

**Keywords:** network anomaly detection, human interaction guidance, network operation, reinforcement learning

## Abstract

With the advancement in big data and cloud computing technology, we have witnessed tremendous developments in applying intelligent techniques in network operation and management. However, learning- and data-based solutions for network operation and maintenance cannot effectively adapt to the dynamic security situation or satisfy administrators’ expectations alone. Anomaly detection of time-series monitoring indicators has been a major challenge for network administrative personnel. Monitored indicators in network operations are characterized by multiple instances with high dimensions and fluctuating time-series features and rely on system resource deployment and business environment variations. Hence, there is a growing consensus that conducting anomaly detection with machine intelligence under the operation and maintenance personnel’s guidance is more effective than solely using learning and modeling. This paper intends to model the anomaly detection task as a Markov Decision Process and adopts the Double Deep Q-Network algorithm to train an anomaly detection agent, in which the multidimensional temporal convolution network is applied as the principal structure of the Q network and the interactive guidance information from the operation and maintenance personnel is introduced into the procedure to facilitate model convergence. Experimental results on the SMD dataset indicate that the proposed modeling and detection method achieves higher precision and recall rates compared to other learning-based methods. Our method achieves model optimization by using human–computer interactions continuously, which guarantees a faster and more consistent model training procedure and convergence.

## 1. Introduction

The modern world has been in the midst of significant changes in network-based informatization and digitalization, amid surges in 5G data transmission, cloud data services, and distributed and federated data computing with machine intelligence. In this context, human-oriented network monitoring, operation, and maintenance approaches have become less able to handle current network administration tasks. Traditional IT operation and maintenance teams are confronted with more complicated network infrastructures, essential services, and online business environments. Applying machine intelligence in IT operations strengthens and optimizes the existing operations and maintenance procedures, with machine learning aiding in specific scenarios where problems cannot be easily identified or solved by manual decisions and operations. AI technology is widely believed to extend human analysis and understanding capabilities for network system monitoring and maintenance with high confidence and low cost.

System observation data in network operation scenarios have different types and presentation formats, including historical data, traffic data, log data, network device status, resource usage data, etc. To be specific, daily real-time monitoring and detection data of network status are mainly network system indicators, such as resource indicators of servers and network devices, online business operation indicators, and system performance indicators. Essentially, these indicators are usually time-series data, which means they are pairs of timestamps with fixed time intervals and data values of the corresponding indicators. Based on the structural characteristics, these different time-series data can reflect the diversified aspects of the network environment’s operations intuitively and inform us about data storage and processing and transmission stability.

It is of great importance to identify the appearance of anomalous time series data during network operation. Network anomaly detection for administration should be able to unveil abnormal fluctuations in system indicators and recognize abnormal network behavior by tracing data variance in time series. Network anomaly detection is an integral part of system monitoring and the underlying core technology of network operations. Any detected abnormal situations may indicate some potential failures in the related services or applications, such as network connection failures, server operation failures, system configuration errors, defective software versions, network resource overloads, server malfunctions, and external attacks.

From an operation perspective, it is a great challenge for the network operation personnel to notice abnormal conditions from the massive amounts of monitored data instantly, especially considering the expansion in system scales, the growth in complexity, and the extension of the monitoring coverage. Under these circumstances, building a network operation model and identifying time series data anomalies with machine intelligence are consistently desired by network administrators and service users. This paper proposes to analyze the fundamental characteristics of time-series data with machine learning by designing a new reinforcement learning procedure on network system indicators with human guidance for anomaly detection agents. The network administration personnel are involved in the learning loop by providing interactive recognitions or rebuttals for the currently monitored network indicators and the latest model-detected anomalies. Such input is fed back into the reinforcement learning iterations to guide the model preference to converge toward the human operators’ focus and interest. We believe a practically effective model for network anomaly detection is more valuable if it can detect not only what the training data imply is anomalous, but what the human operators may feel is wrong.

Overall, the presented interaction-guided learning and detection methodology for network anomalies has theoretical and practical significance regarding the design and development of intelligent network operation and maintenance with human operators in the loop. Humans are the observation supervisors and detection evaluators, and the machine learning procedures are the laborers and followers of the human’s dynamic understanding and scenario interpretation of the network situation. We present our methodology with the following innovations and contributions:

- We manage to utilize a reinforcement learning method to construct network anomaly detectors, which effectively follows the network status dynamics and benchmarks its time-series consistency with past observations and current human supervisions.
- We reconstruct the Temporal Convolution Network (TCN) as the principal structure of the Q-network, which better processes the temporal features in network dynamics and achieves a higher accuracy.
- We introduce human interaction guidance into the learning reward feedback procedure. Users can explicitly provide professional confirmations and evaluations to the currently detected anomalies, which amplify the reward significance for learning model adjustment and convergence.

The rest of the paper elaborates the presented methodology in more detail. Section 2 presents an extensive literature review of the related studies. Section 3 demonstrates the proposed anomaly detection method based on reinforcement learning. Section 4 discusses the proposed human–model interaction mechanism. Section 5 reveals the experiment results and compares the proposed method with other competitors. Section 6 concludes with summarized insights and further discussion.

## 2. Related Work

Generally, network anomaly detection methods fall into two categories, statistic-based and time-series-modeling-based methods. From a data prospective, there are corresponding processing methods for handling different types of data, such as Time Series Decomposition [1] and Holt–Winters [2] for periodic data and fixed threshold, Moving Average [3], Exponentially Weighted Moving Average [4], and Autoregressive Integrated Moving Average [5] for stationary data. Algorithms for non-stationary time series data include Extreme Value Theory and Wavelet [6]. Of course, condensing the huge volume of data into representative features is always challenging and requires feature engineering efforts [7].

Anomaly detection based on statistics is the earliest and the most widely used method. From data distribution, anomalous data samples may deviate from the statistical distribution of normal samples. First, assuming that the given time series follows a certain probability distribution, if the sample cannot fit the model, it is anomalous. Specifically, statistical methods can also be divided into distance-based, density-based, and clustering-based anomaly detection, such as Local Outlier Factor [8], K-Nearest Neighbor [9], Isolated Forest [10], K-Means [11], etc. Li et al. [12] proposed a method for anomaly detection of multivariable time series, focusing on transforming multivariable time series into univariate time series. They applied the Hidden Markov Model (HMM) to detect the abnormal phenomena in multivariate time series and constructed an anomaly detector based on the HMM. To solve the problem that the reliability of cloud services declines with the increase in system complexity, Huang et al. [13] studied a Support Vector Data Description (SVDD) method for detecting abnormal performance indicators of cloud services. Although SVDD has a general anomaly detection ability, it has a high false alarm rate and computational complexity for time series anomaly detection, which seriously affects its practical application. As an improvement, they introduced a simple form of linear programming SVDD. Although the current anomaly detection method based on statistics can be applied to some application scenarios, the disadvantage is that it needs to know the data distribution before detecting anomalies. The distribution is determined by the spatial distance between the data and is not related to the chronological sequence. That is, the statistical method mainly detects the anomalies of unordered data.

Modeling-based anomaly detection methods for time series first build a prediction model. A residual sequence is obtained by comparing the predicted sequence with the observed sequence and is then analyzed to determine whether there is any inconsistency in the original sequence. Malhotra et al. [14] use stacked Long Short-Term Memory (LSTM) for anomaly detection of time series. The network processes time-series data without outlier samples for training. The prediction error is modeled as a multivariate Gaussian distribution and used to evaluate the possibility of an outlier. Malhotra et al. [15] proposed an anomaly detection method for an encoder–decoder based on LSTM. This method first reconstructs a normal time series and then uses the reconstruction error to detect an anomaly. Kim [16] proposed C-LSTM to model traffic data’s spatiotemporal information effectively. They combined a Convolutional Neural Network (CNN), LSTM, and a Deep Neural Network (DNN) to extract more complex features. The CNN layer is used to reduce the frequency variation in spatial information. The LSTM layer is used to model time-series information and the DNN layer maps data to a discrete space.

Although there are various anomaly detection methods for time series, there are not enough benchmarks to test and evaluate their detection performance. Therefore, Lavin et al. [17] proposed Numenta Anomaly Benchmark (NAB), which provides a controlled and reusable open-source tool to test the anomaly detection algorithm series data. The detector, with excellent performance, can quickly detect all anomalies without triggering false alarms, process real-time time series data across multiple domains, and automatically adapt to changing statistical characteristics. These performance metrics are defined and quantified in NAB as scoring algorithms for time series anomaly detection. The Yahoo team [18] designed a general and scalable automatic anomaly detection framework for large-scale time-series data. They used a collection of detection and prediction models with an anomaly filter layer in the EGADS framework to carry out accurate and scalable anomaly detection in time series. Specifically, they chose different anomaly detection methods for each type of time series data. Nevertheless, such a complex system requires users to understand the components of each part and different algorithms so as to adjust each model’s parameters individually.

The above algorithms and tools are general time series anomaly detection methods. Recently, there has been much research efforts and progress on the anomaly detection of time series data in network operation, and they show a trend from methods based on statistics to methods based on machine learning. Xu et al. [19] proposed an unsupervised anomaly detection algorithm, Donut, with a Variational Autoencoder (VAE). The algorithm works without labels at all and can use occasional tags. They further proposed a new understanding of kernel density estimation in z-space, which is helpful for the design of other deep generative models in anomaly detection. Chen et al. [20] pointed out that operation data contain non-Gaussian noise and a complex data distribution, making it difficult to perform time-series modeling. Therefore, they presented a Bayesian network adversarial training method based on partition analysis. Zhao et al. [21] proposed an automatic general anomaly detection framework called Period, which was used to process network operation and maintenance time series data with different periodic characteristics. The core idea is to obtain k subsequences using the proposed sequence clustering technology and then transform the anomaly detection of the unknown period into the anomaly detection of k subsequences with a clear period. However, Period sets the period of the sequence to 1 day by default, making it impossible to effectively process the sequences of other periods. Su et al. [22] proposed a stochastic Recurrent Neural Network (RNN) for multivariable time series anomaly detection called OmniAnomaly. The core idea is to use key technology such as random variable connections and plane standardized flow to obtain the normal pattern by learning the robust representation of multivariate time series, using these representations to reconstruct the input data, and using the reconstruction probability to determine the anomaly. The above methods have achieved good performance in different types of real network operation datasets. However, because they all rely purely on unsupervised learning methods, the selection of a threshold determines the proportion of true and false positives, which potentially impairs the detection effect and corrupts the stability of the algorithms in practice.

Generally speaking, few methods consider the evolution of anomaly patterns against the observation background over time, which leads to the poor performance of static anomaly detection configurations in dynamic scenarios. An ideal anomaly detection model suitable for intelligent operation and the maintenance environment should have the following characteristics. Firstly, no assumption should be made about the anomaly concept, that is, the definition of the anomaly. What is anomalous should only be derived from the training dataset. Secondly, no predefined threshold should be present, which means that the anomaly detection model should be a logic classifier without fixed thresholds. The detector should have no other manually adjustable parameters except the super parameters, for example, the number of layers in a neural network. Finally, the anomaly detection model should continuously improve dynamically with the accumulation of anomaly detection experience. In other words, the detection model can continuously learn new forms of anomalies and enrich its anomaly knowledge base accordingly.

## 3. Learning for Detection

In network management, administrators continuously observe system operation indicators, such as the device resource usage and the link traffic throughput. The characteristics of outliers in such data observations may vary with hardware/software upgrades, system reconfigurations, and the change in service business. It is impossible to maintain the long-term timeliness of outlier detection methods with some fixed thresholds in operation dynamics. As an adaptive control method, reinforcement learning is a viable solution with self-evolution capability. By constructing the anomaly detection process as a Markov Decision Process (MDP), we propose to use Prioritized Experience Replay-based Double Deep Q-Network (PER-DDQN) to evolve anomaly detection models for network anomaly tracing and recognition. This section first presents the core idea and an overview of the MDP and then introduces the anomaly detection and model learning procedure.

### 3.1. Building MDP

**Figure 1:**
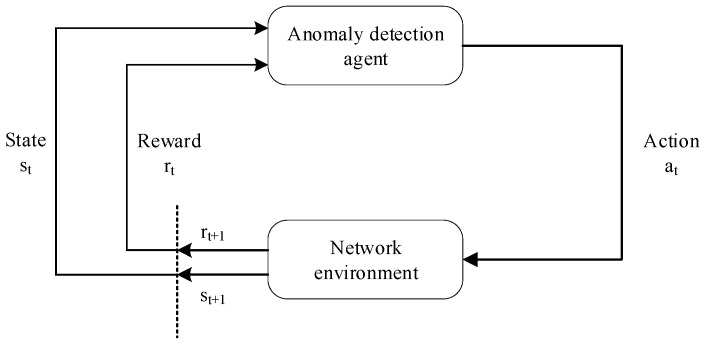
Markov Decision Process for modeling in a network environment.

As shown in Figure 1, in the interaction loops between the anomaly detection agent and the network operation environment, the agent receives the state or observation from the environment at each time interval. According to the state, the agent selects an action (raises an alarm or keeps muted) and executes it. Then, it promotes the transition of the network operation environment state. The agent receives a corresponding reward for its action from the environment reactions for the action. For the anomaly detection problem for network operation, we adopt the MDP formalism. The specific definitions of the MDP are as follows:

(1) State space: S represents the state space, that is, all states that an agent can traverse. $s_{t}\in S,t=0,1,2,\ldots ,T$, which represents the time-series data of the network environment at t. $s_{t}$ reflects the network operation stability and status consistency.

(2) Action space: A refers to action space, namely, all actions that can be taken by an agent. $a_{t}\in A,t=0,1,2,\ldots ,T$, represents the alerting action selected by the agent in the state $s_{t}$, which is equivalent to the classification result.

(3) Reward: R:S×A→R, R is the reward function that maps the states and actions to real numbers, recorded as r(s,a). The reward can be a binary form of positive or negative or a continuous value.

(4) Strategy: the strategy π is the mapping s to a that is a=π(s); the internal model of the agent needs to be continuously updated.

(5) Discount factor: γ∈[0,1] is a discount factor specifying how many immediate rewards are preferred to more distant rewards. Since the anomaly detection task tends to optimize single-step decisions rather than continuous decisions, we naturally prefer a lower discount factor.

(6) Value function: Since the reward is only referred to the evaluation of the current state action, it is impossible to measure and compare the advantages and disadvantages of different strategies. Introducing the value function to represent the expected cumulative reward of strategy π under the current state is of great importance. We have a state value function and a state–action value function, defined as follows.

(1) $$V_{\gamma }^{\pi }(s)={Ε}_{\pi }\left[\sum_{t=0}^{\infty }\gamma^{t}r_{t+1}|s_{0}=s\right]$$

(2) $$Q_{\gamma }^{\pi }(s,a)=Ε\left[\sum_{t=0}^{\infty }\gamma^{t}r_{t+1}|s_{0}=s,a_{0}=a\right]$$

(7) Optimal strategy: After determining the value function concept, the optimal strategy can be expressed by the following formula. In an MDP setting, the goal is to find the optimal policy $\pi^{*}$ which maximizes the expected future discounted reward when the agent selects actions in each state according to $\pi^{*}$.

(3) $$\pi^{*}=\underset{\pi }{argmax}\sum_{s\in S}V^{\pi }(s)$$

### 3.2. Evolving the Detection Model with the PER-DDQN Algorithm

The following section details the process of the prioritized experience replay-based DDQN algorithm when evolving the anomaly detection model.

According to the ε-greedy strategy, a value is initialized to weigh exploration and exploitation in the initial stage. Meanwhile, it is essential to set the ε-decay value to gradually reduce the probability of random action selection so that the detection agent gradually tends to select the optimal action. This value needs to be determined according to the size of the state space and the length of the time step.

To avoid the fluctuation resulting from a single Q-value network, two neural networks with the same parameters are initialized [23]. The principal network is denoted as Q and the target network as $Q^{\prime }$. θ and $\theta^{\prime }$ represent the parameters of the two networks. Simultaneously, the number of iteration rounds C is set. When the number of iterations reaches C, the parameters of the main network are assigned to the target network. In the remaining iteration steps, the target network will not be updated.

In addition, a fixed-size experience pool needs to be initialized. The experience pool is designed to ensure the independence of samples in training. In contrast, if continuous samples are used for random gradient descent training, then there will be a strong correlation among the samples and the variance of parameter updating is large, which makes it difficult to converge. As mentioned above, the experience pool adopts sampling with replacement; another advantage of this method is that the samples can be reused to improve data utilization.

Nevertheless, the benefit of each network observation sample in the experience pool for model updating differs greatly. In network anomaly detection, owing to the sparse positive samples, it is far more important for the agent to learn from the positive samples. In this case, the priority of the sample and sample can be further defined according to the priority [24]. Generally, the Temporal Difference (TD) error is used to represent the priority, and Equation (4) is the calculation formula for the TD error. Priority is usually represented by $p_{t}$, $p_{t}\leftarrow \left|\delta_{t}\right|$. In order to enhance the efficiency of sampling, the data structure of the sum tree is used to store $p_{t}$.

(4) $$\delta_{t}=r_{t}+\gamma {max}_{a}Q(s_{t+1},a_{t+1}|\theta )-Q(s_{t},a_{t}|\theta )$$

The agent explores the state space by selecting random actions or selects the best actions based on the current strategy. Then, the agent executes an action, that is, it makes a judgment whether the observed time-series data in the network environment are normal or abnormal. A human trainer gives the corresponding reward to the agent after receiving the alarm information (agent action decision). Meanwhile, the agent receives the state of the next time step and stores $<s_{t},a_{t},r_{t},{s^{\prime }}_{t}>$ in the experience pool. In order to ensure that every sample can be used at least once when storing in the initial stage, the priority is set to $p_{t}=\max_{i<t}p_{i}$.

When the accumulated samples in the experience pool reach a certain amount, mini-batch-sized samples are collected according to the priority to update the Q-network. The probability of each sample being extracted is $P\left(t^{\prime }\right)=\frac{p_{t^{\prime }}^{\alpha }}{\sum_{t}p_{t}^{\alpha }}$. α indicates the influence of priority on sampling. If it is 0, it will degenerate into uniform distribution sampling. The priority experience replay alters the distribution of the state space, so the deviation is incorporated in the update. It is necessary to set the importance sampling weights to correct the deviation. Equation (5) presents the calculation of weight.

(5) $$w_{t^{\prime }}=\frac{{\left(N\times P(t^{\prime })\right)}^{-\beta }}{{max}_{t}(w_{t})}$$

After adding the weight of correction deviation, the loss function is defined as follows.

(6) $$\frac{1}{m}\sum_{i=1}^{m}w_{t}(y_{t}-Q(\phi (s_{t}),a_{t}|\theta ){)}^{2}$$

**Figure 2:**
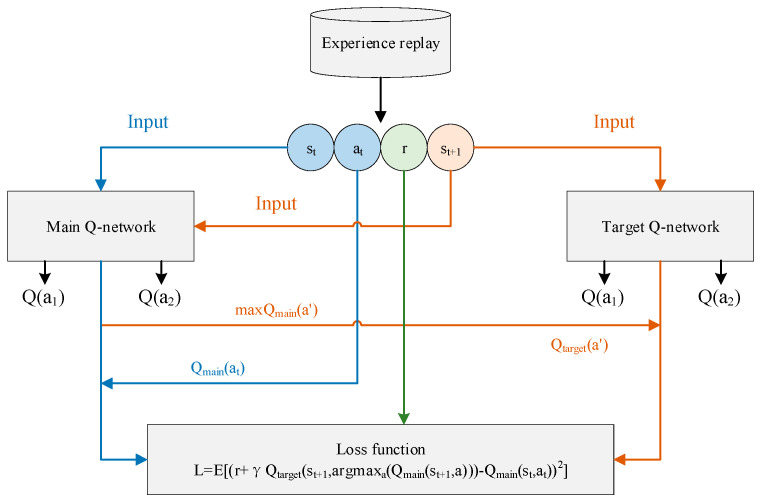
Double Deep Q-Network algorithm.

The overestimation of the Q value in a DQN is eliminated by a decoupled selection of a target Q value action and the calculation of a target Q value. Figure 2 shows the detailed update process. The final target Q value is obtained by Equation (7).

(7) $$y_{t}=r_{t}+\gamma Q^{\prime }(\phi ({s^{\prime }}_{t}),argmax_{a^{\prime }}Q(\phi ({s^{\prime }}_{t}),a|\theta )|\theta^{\prime })$$

According to the mean square error function, the parameters of the Q-network are updated by backpropagation. Then, we use the updated network to recalculate the TD error to update the sample priority of the experience pool. If the number of iteration rounds reaches C, further synchronization of the parameters is required.

### 3.3. Q-Network Structure

A TCN is a time series modeling framework based on a convolutional neural network [25]. Compared to WaveNet [26] proposed by DeepMind, the gate mechanism is removed and a residual structure is added. At present, although RNNs have a good performance in most sequence problems, there are some disadvantages in the internal design of RNNs, such as the network can only read and parse one-step data at each time. Actually, RNNs cannot perform parallel processing like CNNs, which regard the sequence as a one-dimensional object and obtains enough receptive field through a multi-layer network structure.

**Figure 3:**
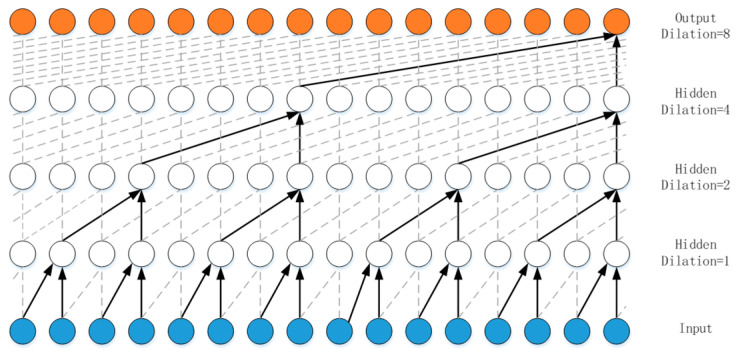
Architecture of dilated convolution.

A TCN uses causal convolution to ensure that the output value does not depend on future information. When calculating the output at t, it can be obtained from inputs no later than t. It is a strict time constraint model and belongs to a unidirectional structure, not a dual-direction structure. Simple causal convolution still has the problem of traditional convolution neural networks, i.e., the size of the convolution kernel limits the modeling length of time. Dilated convolution introduces a new hyperparameter based on ordinary convolution, the dilation factor. Dilation causal convolutions allow the filter to be applied over an area larger than its length by skipping input values with a certain step. For a one-dimensional sequence input $x\in R^{n}$ with filter f:{0,…,k−1}→R, the output can be expressed as:

(8) $$F(t)=(x\times_{d}f)(t)=\sum_{i=0}^{k}f(i)\cdot x_{t-d\cdot i}$$

where d is the dilation factor, k is the convolution size, and t−d⋅i means that only the past state is convoluted. This expansion is equivalent to adding a fixed step size between two adjacent filters in each layer. When the dilation factor is set to 1, the dilated convolution degenerates into ordinary convolution. Using a more extensive dilation factor can effectively make the output of the higher layer represent a broader range of input information to expand the receptive field of the convolutional network. When k=2 and the dilation factor of the hidden layer is [1,2,4,8], as shown in Figure 3, each node in the output layer can see 16 units in the input layer.

The size of the receptive field depends on the network depth, filter size, and dilated factor. In some cases, it is still necessary to increase the network depth to obtain enough receptive field. A residual connection is an effective way to train a deep network, making the network transmit information in a cross-layer way. The residual block contains a series of transformations for the input, adds its output to the input, and activates it.

## 4. Interaction during Detection

It is feasible for the agent to interact with the environment autonomously based on the reinforcement learning algorithm. However, two principal issues exist in such a process. For one thing, under the premise of computing hardware limitations, the sampling efficiency of the reinforcement learning algorithm is relatively low, leading to a slow convergence speed. Additionally, the deviation in the final concept between model construction and the requirements of operation personnel may result in false alarms and omissions. Therefore, a more effective strategy is to speed up the model’s convergence process under guidance from external operators in a specific state.

**Figure 4:**
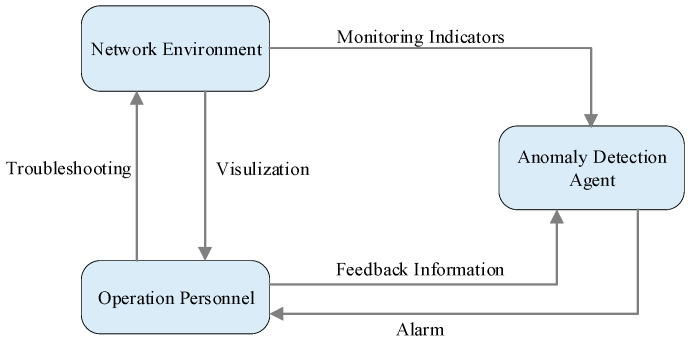
Framework of interactive anomaly detection.

In this section, we propose an anomaly detection framework for network environment management with interactive guidance from human operators. As demonstrated in Figure 4, the framework comprises three parts: a network operation environment, operation personnel, and an anomaly detection agent. The core part of the interaction is the guidance from the operation personnel to the anomaly detection agent. Compared to the traditional interaction between the agent and the environment, the greatest emphasis here is on the human–computer interaction to improve the performance of anomaly detection. Obviously, the human evaluation on the agent’s action is more prominent and directional as a reward for model convergence than the environment reward.

The network operation environment represents the whole network system’s dynamic running, including the hardware, software, and data resources supporting normal network services. At each time step, the network operation environment sends the real-time monitoring indicators to the anomaly detection agent as state vectors and displays the network status to the operation personnel through the network operation monitoring system. Simultaneously, the network operation environment feeds back according to key performance indicators, such as response time, throughput, and concurrency.

Operation personnel are the decision makers and executors of the network operation system. They can ensure the network’s proper functioning by monitoring the network environment and handling the faults in a timely manner. At each time step, the operation personnel can view the time sequence monitoring indicator in the real-time network and the anomaly detection results returned by the agent through the user interface. Operation personnel evaluate the accuracy of alarm information based on historical experience and professional knowledge to confirm or cancel the alarm. If necessary, they also need to carry out fault handling. Operation behaviors are converted into feedback signals to guide the anomaly detection agent to perform specific actions in different states.

Generally, if the agent produces false alarms or underreporting, the operation personnel should give negative feedback signals. If the network status outliers are correctly identified, operators signal positive feedback. Considering the human cost, the interaction between the operation personnel and the agent can be considered an occasional process. In particular, when the operation personnel fail to give immediate feedback, the agent’s anomaly detection results are accepted by default.

The anomaly detection agent is an effective tool to detect outliers from the time dimension. In the proposed framework, the interaction is the process in which the operation personnel guide the agent to learn a target strategy. During this process, the operation personnel deliver this strategy through interaction, where in parallel, the agent learns and integrates it from the feedback.

### 4.1. Active and Passive Interaction

**Figure 5:**
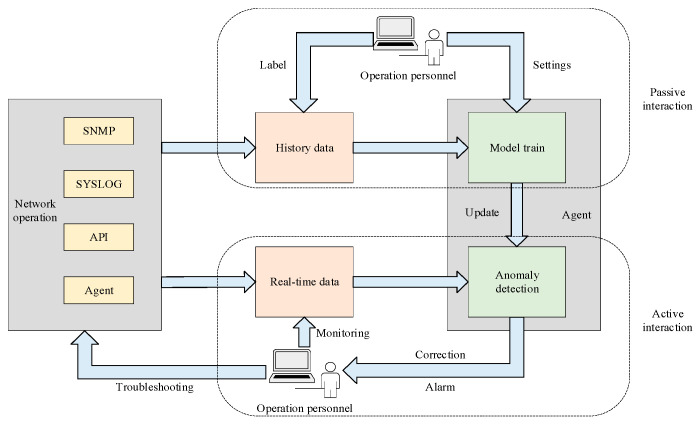
Process of active interaction and passive interaction.

In this framework, human–computer interaction is a two-orientation process, which can be accessed through the user interface in the network operation monitoring system. In terms of the agent’s standpoint, human–computer interaction can be divided into active interaction and passive interaction. Figure 5 presents the detailed process.

Taking the agent as the starting point, active interaction outputs the execution results or suggestions to the operation personnel without instructions. In this case, the agent can extract the time series data and analyze the data to make decisions. In terms of a malfunction, the agent sends an alarm to the operation personnel. However, it is common that humans make mistakes, such as failing to confirm the alarm in a timely manner or mistakenly marking historical records. Under these circumstances, the agent is required to be equipped with the correction ability to assist the judgement of operation personnel. To provide more reliable data support, agents tend to compare multiple similar sequences based on historical records and select the most tagged category as the corrected result.

Passive interaction refers to the process in which operation personnel intervene to interact with the agent to maintain the network environment. In some cases (service adjustment or system update), the temporal characteristics of network status indicators change significantly. Thus, the agent cannot effectively detect and signal abnormal conditions, resulting in a large number of false alarms and underreporting. Based on the observation and collection of real-time monitoring information, the operation personnel can label historical records to correct the missing and false alarms. Additionally, operation personnel can set some specific model parameters and rules for specific service scenarios and performance requirements, such as the sliding window size, interval step, the setting of the reward function, the size of the weight factor, the alarm setting level, etc. Operation personnel directly adjust the model effect through these user interfaces.

### 4.2. Empirical Reward and Objective Reward

In the proposed anomaly detection model, the reinforcement learning agent receives two rewards from different sources, namely, the reward based on the fluctuation of key performance indicators and the reward based on historical experience and professional knowledge. According to their different attributes, the former is defined as an objective reward $r^{E}$ and the latter as an empirical reward $r^{H}$. Considering that the value ranges of the two rewards are different, for example, the fluctuation in network performance indicators is primarily concentrated in [0,0.1], and the feedback of operation personnel can be any continuous value, it is necessary to scale the two rewards to the same range before calculation. The final reward function is calculated by Equation (9) and is adopted to update the Q-network.

(9) $$r(s,a)=\beta \cdot r^{H}(s,a)+(1-\beta )\cdot r^{E}(s,a)$$

In the above formula, β is a predefined parameter, which is applied to assign different weights to the objective reward and the empirical reward. This parameter decreases along with the gradual convergence of the model. With the improvement in the accuracy of agent anomaly detection, the weight of the empirical reward decreases, while the weight of objective reward increases. As a result of sparse abnormal samples, the guidance of operation personnel can help the anomaly detection agent avoid accumulating a large number of invalid training samples in the initial stage. With the deepening of the learning process, agents can gradually infer the guidance intention of operation personnel through interaction.

### 4.3. Reward Mechanism Based on Strategy Evaluation

The empirical reward refers to subjective judgment. How to give it a clear reward mapping to interactive guidance is of great importance. Existing algorithms assume that the reward merely depends on the agent’s action choice. This paper proposes that it also depends on the agent’s current strategy. The reward or punishment after an action selection depends on agent’s action accuracy. It can be explained that the operation personnel tend to give more positive feedback when the strategy of the agent is significantly improved, and when the agent makes mistakes in the near-optimal and relatively stable strategy, it will impose greater punishment on the agent. In this paper, we propose a reward mechanism based on strategy evaluation. The central issue is to give the corresponding reward according to the current strategy evaluation of the agent.

The pivotal part is how to describe the performance compared with that under the current strategy. According to the description of the MDP in the previous chapter, the state value function is equivalent to the long-term cumulative reward expectation in the current state, while the action–value function refers to the evaluation of action selection in the current state. The difference between the two values just reflects the comparative advantage of action selection based on the current strategy. Therefore, as shown in Equation (10), this paper uses the advantage function as the evaluation metric of strategy, and it is also the mapping of empirical rewards.

(10) $$A^{\pi }(s,a)=Q^{\pi }(s,a)-V^{\pi }(s)=r^{H}(s,a)$$

where $A^{\pi }(s,a)$ represents the advantage of an action relative to the average reward in state s. The advantage function normalizes the Q value as a baseline index, which improves the learning efficiency, makes learning more stable, and reduces variance. In the early stage of learning, due to the limited state space exploration, $V^{\pi }(s)$ is at a low level. Therefore, the agent obtains more rewards to motivate it to achieve a better performance. With the gradual optimization of the strategy, the value of the advantage function will become zero or negative. Finally, the advantage function determines whether the action is guided by a positive strategy or a negative strategy according to whether the choice of the action is improved relative to the current behavior to achieve the goal of the learning strategies. In the interaction process, the agent obtains the guidance intention according to the command of the operation personnel and then calculates the value of the empirical reward according to the advantage function.

Generally, the state value function can be approximately calculated in the reinforcement learning algorithm based on strategy gradient, but the reinforcement learning algorithm based on the value function used in this paper cannot directly obtain $V^{\pi }(s)$. In the current stage of intelligent operation development, the agent cannot wholly replace the operation personnel to judge and make decisions, so the anomaly detection agent can only monitor and alarm in complex scenes and cannot directly affect the state change of the network environment. Based on the above description, the anomaly detection task is similar to the typical concept scenario of contextual bandit in reinforcement learning, that is, the change in state does not depend on the previous state or action. Therefore, without considering the long-term cumulative reward, the discount factor is approximately 0 and $V^{\pi }(s)$ can be obtained from Equation (11).

(11) $$V^{\pi }(s)=P(a_{0})r(s,a_{0})+P(a_{1})r(s,a_{1})$$

where $a_{0}$ means silence and $a_{1}$ means alarm. There are two types of hypothetical states: normal, $s_{0}$, and abnormal, $s_{1}$. The reward is set in Table 1. Then, $V^{\pi }(s)$ can be further calculated by Equations (12) and (13).

(12) $$V^{\pi }(s_{0})=\frac{TN}{TN+FP}\cdot (1)+\frac{FP}{TN+FP}\cdot (-1)$$

(13) $$V^{\pi }(s_{1})=\frac{FN}{TP+FN}\cdot (-5)+\frac{TP}{TP+FN}\cdot (5)$$

In this paper, a positive sample refers to an anomaly, and a negative sample refers to a normal state. Accordingly, True Positive (TP) indicates the number of correctly detected anomalies. False Positive (FP) indicates the number of incorrectly classified normal data. True Negative (TN) indicates the number of correctly detected normal data. False Negative (FN) indicates the number of anomalies wrongly classified as normal.

The calculation of the final advantage function is shown in Table 2. When the agent adopts the worst strategy, the accuracy of the model is 0, and the operation personnel tend to give more rewards to make the model converge faster. In the process of strategy optimization, the positive and negative rewards change accordingly. Under the optimal strategy, that is, the accuracy of the model is 1, the operation personnel tend to impose more penalties so that the behavior of the agent can be adjusted. In general, the reward mechanism of strategy evaluation can guide the agent from the perspective of the operation personnel in dynamic strategy optimization.

### 4.4. Correction Mechanism Based on DTW

The interaction module is designed to provide human feedback as a reward to the detection agent, so that the model being trained is guided to converge faster according to the operation personnel’s preference. There are inevitably some human errors in network operation and abnormal records are labeled incorrectly. Therefore, the anomaly detection agent should also have a specific mechanism to distinguish and correct the behavior of operation personnel. In this paper, we utilize the Dynamic Time Warping (DTW) algorithm [27] to handle occasional inconsistent human feedback. When a detection result from the agent is inconsistent with the judgment from the operation personnel, the agent uses the labels of similar historical sequences to mark the ambiguous sequence.

With two sequences, $X=x\left[1\right],x\left[2\right],\ldots ,x\left[i\right],\ldots ,x[n]$, $Y=y\left[1\right],y\left[2\right],\ldots ,y\left[j\right],\ldots ,y[m]$, X and Y can be arranged in a grid of n×m, where each point (i,j) is the distance between x[i] and y[j]. The regular path W is used to map data points in X and Y sequences to minimize the distance. W consists of a series of grid points (i,j).

The optimal path between $\left(i_{k},j_{k}\right)$ is calculated as following:

(14) $$D_{\min}(i_{k},j_{k})=\underset{i_{k-1},j_{k-1}}{min}D_{\min}(i_{k-1},j_{k-1})+d(i_{k},j_{k}\left|i_{k-1},j_{k-1}\right.)$$

where d is the Euclidean distance, so the final similarity can be calculated by the following formula. The smaller the DTW distance is, the more similar the two sequences are.

(15) $$D=\sum_{k}d(i_{k,}j_{k})$$

The DTW algorithm uses the dynamic programming method to align the two sequences to obtain the regular path. However, it takes a lot of time to traverse all possible paths. In order to improve the efficiency, the number of possible regular paths must be limited.

In this paper, ambiguous sequences are recorded as target sequences and historical sequences are recorded as matching sequences. To further reduce the computational overhead, the length of the matching sequence is equal to the length of the target sequence, that is, n=M. A fixed similarity threshold is preset to eliminate dissimilar historical sequences and avoid impacting the results. Finally, based on the labels of similar matching sequences, the correction result of the target sequence is determined by the voting method and returned to the operation personnel for confirmation again.

## 5. Experiments and Evaluation

This section evaluates the performance of the proposed anomaly detection method. We first introduce the datasets, metrics, and settings. Then, the performance of the proposed model compared to the current state-of-the-art schemes for anomaly detection is demonstrated. Finally, we present the effectiveness of the interaction-based guidance mechanism. The experiments are implemented using an i5-9400F CPU @ 2.90 GHz with 16 GB of RAM on Python 3.7 and TensorFlow 2.1.

### 5.1. Datasets

We conducted our experiments on one-dimensional Key Performance Indicators (KPI) and multidimensional Server Machine Dataset (SMD). We list the summary statistics for the datasets in Table 3 and Table 4.

The detailed information of the datasets is described in the following.

#### 5.1.1. KPI

**Figure 6:**
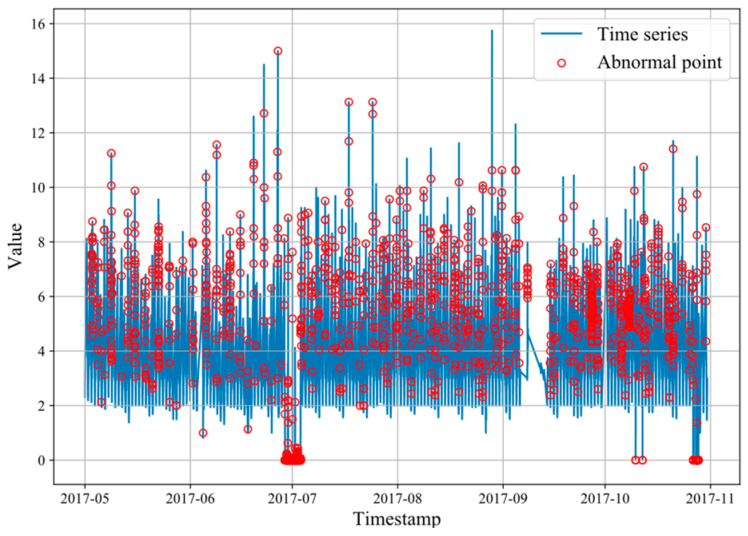
Sequence diagram of KPI.

The KPI dataset is from NetMan Laboratory [28]. The dataset consists of multiple KPI curves with anomaly labels collected from various internet companies, including Sogou, Tencent, eBay, etc. Most KPI curves have an interval of 1 min between two adjacent data points, while some have an interval of 5 min. The dataset includes 29 different monitoring indicators. To verify the ability of the model to detect different types of anomalies, dataset no. 6 was selected as the experimental dataset. As shown in Figure 6, the dataset contains outliers, contextual exceptions, and collective anomalies.

#### 5.1.2. SMD

The SMD is a 5 week long dataset that was collected from a large internet company. This dataset contains three groups of entities and is made up of data from 28 different machines. We conduct our experiments on the <machine-1-1> dataset. It was divided into two subsets of equal size; the first half is the training set and the second half test set. Domain experts have labeled anomalies and their anomalous dimensions in the SMD testing set based on incident reports. The data collection time interval is 1 min. As shown in Table 4, there are CPU load, network usage, and memory usage records for each machine record, including 38 dimensions.

### 5.2. Metrics

The following metrics were applied to evaluate the performance of the proposed approach.

In Equation (16), accuracy represents the proportion of samples correctly classified.

(16) $$\mathrm{Accuracy}=\frac{TP+TN}{TP+TN+FP+FN}$$

In Equation (17), precision refers to the proportion of correctly classified anomalies relative to all samples classified as an anomaly. The higher the precision, the lower the false alarm rate.

(17) $$\mathrm{Precision}=\frac{TP}{TP+FP}$$

In anomaly detection, recall is the most crucial evaluation indicator, representing the proportion of anomalies detected to all abnormal data (Equation (18)). A higher recall comes with a lower missing report rate.

(18) $$\mathrm{Recall}=\frac{TP}{TP+FN}$$

One of the most common anomaly detection issues is that it is hard to achieve a high precision and recall simultaneously. In this case, the F1-score measures the harmonic mean of precision and recall, which can comprehensively reflect the performance of the anomaly detection model.

(19) $$F1\text{-}\mathrm{score}=\frac{2\times \mathrm{precision}\times \mathrm{recall}}{\mathrm{precision}+\mathrm{recall}}$$

### 5.3. Parameter Settings

#### 5.3.1. Reinforcement Learning Settings

We can adjust the model’s detection attention to the historical observations by tuning the discount factor γ, which determines the tradeoff between attention and the long-term or short-term returns. With a γ value closer to 0, less future rewards are counted. With γ=1, all future rewards are considered in the calculation of current state–action value function. Since the anomaly detection task in a network traffic detection scenario is more concerned with current abnormal conditions, we set γ as 0.01 in the following experiments.

The length of the input vector of the Q-network was set to 60. The dilations of each layer in TCN were set to [1, 2, 4, 8, 16, 32] and the number of convolution layer filters was 64. The loss function of the model was calculated as the mean square error with modified deviation weight, and the optimizer was Adam.

#### 5.3.2. Interaction Settings

In the interaction process, the operation personnel are modeled with multiple attributes as simulation parameters. Specifically, interaction frequency, persistence, and accuracy are used to represent the interaction behavior of the operation personnel.

The interaction frequency refers to the proportion of data that operation personnel interact with among all the samples. In the process of active interaction, most of the alarm information contains a continuous number of abnormal samples, and the operation personnel can confirm or negate them in batch, where the interaction frequency is 100%. In the process of passive interaction, that is, when checking and tagging the historical records, the operation personnel have to deal with most of the normal time-series data, in which the abnormal points are very sparse, so the interaction frequency is 0.1%.

Interaction persistence refers to the duration steps of operation personnel interaction. Considering that the operation personnel cannot give the agent feedback information continuously due to various objective conditions, the duration steps are generally set to 5. The experience replay mechanism is used in the anomaly detection model; thus, the attribute will not affect the detection performance of the agent.

Interaction accuracy refers to the correctness or quality of the feedback information from the operation personnel. As mentioned previously, during the instant monitoring and management operations, administration personnel may inevitably make some mistakes. Such false information may lead to some deviation of the agent’s understanding of the anomaly concept. In this paper, the proportion of operation personnel’s misjudgments in all the interaction samples is simulated with a setting of 0.1%. These samples are used to verify the active correction mechanism based on DTW in the experiment, and the approximate distance threshold is set to 5.

### 5.4. Results and Analysis

#### 5.4.1. Results on the Training Set

**Figure 7:**
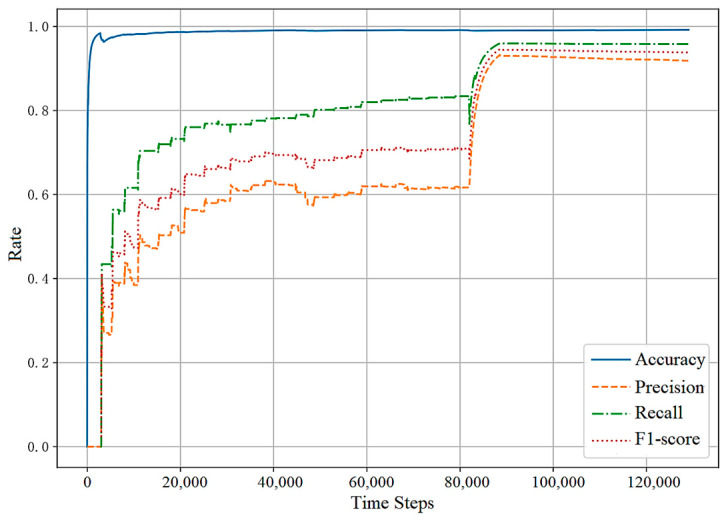
KPI training set result.

**Figure 8:**
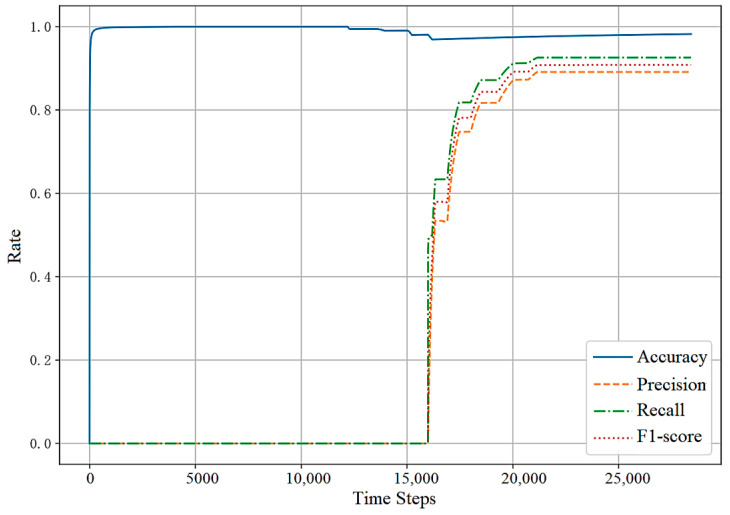
SMD training set result.

Figure 7 and Figure 8 show the changes in the evaluation metrics of the training set as the time increases. Due to the method of incremental learning, the value of the evaluation metrics is cumulative. A large number of FPs and FNs generated at the initial stage of training are also included in the calculation of metrics. As can be seen from the figures, compared with other evaluation metrics, the accuracy rate increases fastest and is always close to 0.99. This is due to the extreme imbalance of the dataset and the sparse existence of anomalies. In addition, in the reward function setting, a larger reward and punishment are given to the action selection of abnormal samples, resulting in the recall rate always being greater than the accuracy.

In Figure 7, the F1-score at the initial stage of training is below 0.6. The reason lies in the fact that the anomaly detection agent does not initially have any prior knowledge about the traffic situation, leading to high randomness in its action selection. With the accumulation of anomaly detection experience, the detection orientation and performance of the agent significantly improves. Due to the occurrence of collective anomalies, the metrics promptly climb after 80,000 steps. In Figure 8, since the first abnormal point of SMD appears after 15,849 steps, the precision, recall, and F1-score before are all null values. Similarly, with the accumulation of abnormal samples, the F1-score of the model is gradually improved.

#### 5.4.2. Results on the Test Set

To demonstrate the effectiveness of the proposed method, we first compared it with four state-of-the-art approaches: Donut 18, Buzz 19, Period 20, and OmniAnomaly 21. Table 5 shows the precision, recall, and F1-score of different methods on both datasets. Our proposed method outperforms the others on both KPI and SMD datasets, with a superiority over the best performing state-of-the-art method by 0.02 and 0.04, respectively, in the F1-score for both datasets. The precision and recall of the proposed method are higher than 0.9 for both datasets, which is not achieved by other baseline approaches. Overall, these experimental results demonstrate the superiority of the proposed anomaly detection method compared with state-of-the-art approaches.

**Figure 9:**
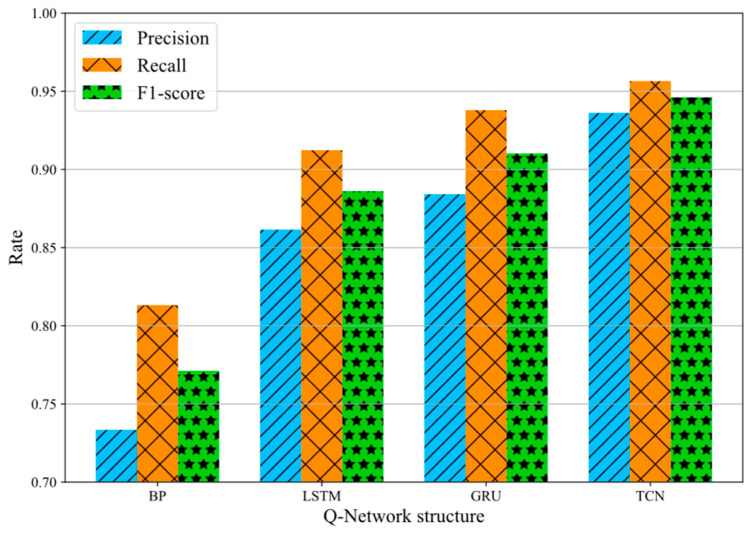
Performance comparison of different Q-network structures.

Next, we used different neural network structures as the Q-network, including a DNN, LSTM, GRU, and a TCN. Figure 9 shows the total precision, recall, and F1-score obtained by different Q-network structures for the KPI and SMD datasets. It can be seen from Figure 9 that the TCN is better than other neural networks in anomaly detection tasks because it has a flexible receptive field size and stable gradients.

#### 5.4.3. Results of the Interaction Mechanism

**Figure 10:**
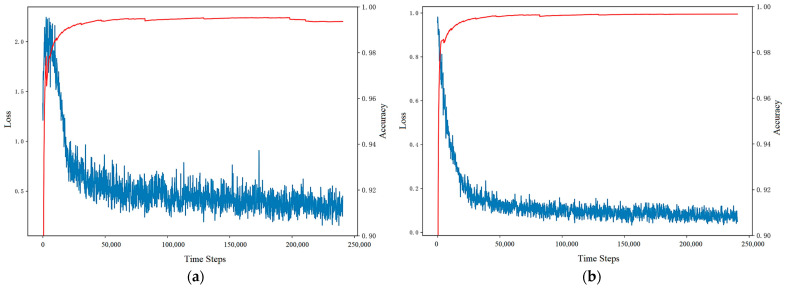
Loss and accuracy curve for: (**a**) the non-interactive model; (**b**) the interactive model.

In this part, we compared the non-interactive model and the interactive model. For the convenience of analysis and display, we conducted a contrasting experiment on the KPI dataset. Figure 10 shows the loss and accuracy curves in the training process. For the anomaly detection model without interactive guidance, the loss increases first and then decreases in the initial training stage. After about 50,000 steps, the model converges gradually, but the loss still presents considerable volatility, and the final loss is around 0.4. As for the model with interactive guidance, we can see that the loss curve drops very fast after adding the empirical reward given by the operation personnel, and the loss drops to less than 0.2 within 50,000 steps, which indicates that the interaction mechanism proposed in this paper can effectively accelerate the convergence speed of the anomaly detection model. Additionally, due to the use of a reward mechanism based on strategy evaluation, the model’s performance is more stable. Compared with the non-interactive model, the fluctuation of the loss is smaller, and it is reduced by about 50%.

As shown in Table 6, the interactive model shows significant improvements in evaluation metrics compared with the non-interactive model. The precision, recall, and F1-score of the interactive model are improved by up to 0.03. In addition, it can be seen that the precision and recall are closer under the guidance of operation personnel, which indicates that the empirical reward can effectively balance the tradeoff between the two metrics.

In the experiment, based on the 0.001 misjudgment probability of operation personnel, nine misjudged samples were generated. These samples are all underreported samples. Abnormal points are recorded as normal data by operation personnel. For all the misjudged samples, the agent will use the active correction mechanism based on DTW to proofread. Table 7 shows the results of the correction mechanism. The number of the target sequence, the number and similarity of the most similar sequence, the number of samples below the similarity threshold, and the correction results are listed in the table. In the calculation of similarity, we also follow the principle of temporal relations by only comparing with the past sequence. It can be seen from the table that the success rate of active correction is 100%, avoiding detection performance degradation caused by the misjudgment and correcting the underreporting records in time to reduce the loss.

## 6. Conclusions

This paper presents a robust anomaly detection method based on reinforcement learning and an interaction mechanism in network operation, particularly for monitoring indicators. By combining prioritized experience replay and the DDQN algorithm, reinforcement learning is applied to network anomaly detection tasks in parallel with network operations. To achieve better results on time series data, we propose to incorporate a TCN as the principal structure of the Q-network. Additionally, we further design an anomaly detection framework with interactive guidance from operation personnel. We give clear definitions for the concepts of active interaction and passive interaction during the interaction process. In particular, the combination of objective and empirical rewards and a reward mechanism based on strategy evaluation are proposed for the agent’s internal update. We adopt the DTW active correction mechanism to correct false guidance information produced by the operation personnel. Experimental results on the KPI and SMD datasets demonstrate that the proposed method outperformed other anomaly detection techniques and the interaction mechanism can effectively accelerate the convergence speed of the model.

While the current system architecture and detection procedure are complete, there are still some limitations in the proposed approach, which we are investigating further to make effective improvements. First, we need a more effective way to condense the hyper-dimensional network traffic time-series data to present the current network situation to the administration personnel. For instant monitoring, humans can only percept and understand traffic data with proper sampling and forensic representations. Secondly, current human–model interactions are modeled as data labeling with uncertainty. Although we have considered a scenario with human error, the validation and correction mechanisms require more precision and efficiency. Additionally, thirdly, adapting the proposed mechanism with existing online traffic surveillance and detection systems such as IDS/ADS is desirable, especially when the traffic volume in a realistic system could be too much to completely undergo the current modeling and detection procedure. For the data analysis and processing parts, we plan to gain further insights into the method optimization of time series classification so that we can design different anomaly detection methods for diversified service requirements. Furthermore, we expect to build up more in-depth knowledge about the interaction mechanism to probe effective methods for human–agent interactions. While this presented work shows promise for effective combination of computer intelligence and human guidance, we look forward to more sustainable, adaptable, and systematic solutions for applying such mechanisms in realistic network traffic detection and analysis in the near future.

## Figures and Tables

**Table 1:** Reward function.

	Predicted	Normal	Anomaly
Actual			
Normal		1	−1
Anomaly		−5	5

**Table 2:** Calculation of the advantage function.

Strategy	State	$V^{\pi }(s)$	$A^{\pi }(s,a)$	
			$a_{0}$	$a_{1}$
Best	$s_{0}$	−1	2	0
	$s_{1}$	−5	0	10
Worst	$s_{0}$	1	0	−2
	$s_{1}$	5	−10	0

**Table 3:** Statistics of datasets.

Datasets	Duration	Sample No.	Anomaly No.	Anomaly Ratio (%)
KPI	6 months	240,612	11,508	4.78
SMD	5 weeks	56,958	2694	4.72

**Table 4:** Dimensions of the SMD.

Types	Dimensions
CPU	cpu_r, load_1, load_5, load_15
Memory	mem_shmem, mem_u, mem_u_e, total_mem
Disk	disk_q, disk_r, disk_rb, disk_svc, disk_u, disk_w, disk_wa, disk_wb, si, so
Network	eth1_fi, eth1_fo, eth1_pi, eth1_po, tcp_tw, tcp_use, active_opens, curr_estab, in_errs, in_segs, listen_overflows, out_rsts, out_segs, passive_opens, retransegs, tcp_timeouts, udp_in_dg, udp_out_dg, udp_rcv_buf_errs, udp_snd_buf_errs

**Table 5:** Performance comparison of the proposed model with state-of-the-art techniques.

Method	KPI			SMD		
	R (%)	P (%)	F1(%)	R (%)	P (%)	F1(%)
Donut	87.32	85.24	86.27	82.35	81.32	81.83
Buzz	93.34	91.62	92.47	82.34	82.13	82.23
Period	92.57	92.80	92.68	82.31	83.85	83.07
Omni Anomaly	97.76	88.67	92.99	94.49	83.34	88.57
Proposed	95.65	93.61	94.62	93.31	92.33	92.82

**Table 6:** Performance comparison of the non-interactive model and the interactive model.

Models	Accuracy (%)	Recall (%)	Precision (%)	F1 (%)
Non-interactive	99.36	93.68	92.96	93.32
Interactive	99.67	96.22	95.77	96.00

**Table 7:** Results of the correction mechanism.

Sample No.	Similar Sample No.	Abnormal Sample No.	No. of Most Similar Sample	Similarity	Correction Result
55,746	26	26	17,916	1.5245	anomaly
84,821	37	37	83,359	0	anomaly
99,125	48	48	11,093	1.6441	anomaly
100,370	13	13	20,769	2.3452	anomaly
143,951	37	37	38,122	0.6884	anomaly
180,615	54	54	170,721	1.3904	anomaly
196,273	33	33	48,739	2.5627	anomaly
217,057	87	87	135,467	1.1128	anomaly
223,719	26	26	20,761	1.0675	anomaly

## Data Availability

The KPI and SMD datasets used for the experiments in this paper are publicly available online at the providers’ websites. The processed results can be obtained from the corresponding author upon request.
